# Rehabilitation coordinator intervention versus control in psychiatric specialist care for return to work and reduced sick leave: study protocol of a randomised controlled trial

**DOI:** 10.1186/s12889-020-8238-3

**Published:** 2020-02-19

**Authors:** Åsa Andersén, Erik Berglund, Ingrid Anderzén

**Affiliations:** Department of Public Health and Caring Sciences, Uppsala University, P.O Box 564, SE-751 22 Uppsala, Sweden

**Keywords:** Return to work, Rehabilitation, Coordination, Specialist psychiatric care, Intervention

## Abstract

**Background:**

Mental disorders are the most common reason for sick leave in Sweden. Knowledge about effective methods to help these individuals to return to work (RTW)/entry into work or studies is limited. Rehabilitation coordinators (RC’s) have been introduced within healthcare with the purpose to promote cooperation, streamline the sick leave and rehabilitation process, and facilitate RTW for sick-listed patients. The function of RC’s has shown positive results by reducing sick leave within primary healthcare. However, the function has not been evaluated in terms of specialist psychiatry. This paper describes the design of a study to evaluate effects of a RC intervention on sick leave and RTW/entry in work or studies in patients with moderate to severe affective and/or moderate to severe anxiety disorders within specialist psychiatric care.

**Methods:**

A randomised controlled trial (RCT) comparing an intervention group receiving support from a RC with a control group receiving treatment as usual (TAU). The target group is patients on sick leave, treated for affective and/or anxiety disorder, aged 25–64, with or without employment.

**Discussion:**

This study gives the possibility to evaluate a RC intervention for individuals with mental disorders. If the study has promising vocational outcomes, it may be of importance for the participants in many ways, e.g. increase participation in society, provide economic benefits and improve health and wellbeing. This would be valuable for the individual as well as for the society.

**Trial registration:**

The study is registered at the Clinicaltrials.gov Register Platform (ID NCT03729050) in 2 November 2018.

## Background

Mental disorders are a major challenge in Europe, with depression and anxiety being the most common [1]. In Sweden, mental disorders are also the most common reason for sick leave [2]. Sick leave may be necessary for healing and recovery and prove to be necessary due to the tasks required for the specific work. However, longer periods of sick leave by itself can have negative impact, including impaired health [3, 4] and reduce the possibility for return to work (RTW) [5]. Individuals with mental disorders are in general more likely to be unemployed [6]; moreover, among young individuals diagnosed with depression- or anxiety disorder (bipolar disorders not included), 20% were still outside of the labour market 10 years later [7]. Similar to long-term sick leave, unemployment at young age has also been associated with negative effects on health and well-being [8, 9]. This negative impact on health may further complicate the possibility of entering the labour market, which in a long-term perspective may lead to marginalisation and exclusion from the labour market and society for these individuals. Nonetheless, knowledge about what actions are effective in supporting individuals with mental disorders to RTW/entry work or studies is limited [10].

Longer durations of sick leave (≥60 days) increase the need for vocational rehabilitation [11], which is rehabilitation interventions aimed at facilitating RTW [12]. Hence, individuals suffering from injury or illness have the possibility to receive rehabilitation and support to get prerequisites to regain work ability [13]. Several actors can be involved in the vocational rehabilitation e.g. the Swedish Social Insurance Agency (SSIA), healthcare, employers, Swedish Public Employment Service (SPES) and the municipality [14], each having their own responsibilities [14]. Work ability can be judged differently among the actors involved in the vocational rehabilitation, which can lead to contradictions between them [15] and thus constitute an obstacle in the rehabilitation process. To reach positive RTW outcomes, it is assumed that cooperation between these actors and the individual takes place [16]. Lack of cooperation between these actors can instead complicate the individual’s ability to RTW [17]. Factors that influence the individual’s opportunity to RTW successfully include participation from the employer and support from the workplace [10]. Moreover, contact between the healthcare and the employer as well as involvement of a person who coordinates the vocational rehabilitation are important [18].

A previous meta-analysis demonstrates a clear but moderate effect on the RTW for employees who were on sick leave and who had access to a person who coordinated their vocational rehabilitation process [19]. Nonetheless, another review could not show that RTW coordination was more effective than usual practice with respect to RTW [20]. However, the RTW coordination can be carried out differently with regard to content, length and design.

In Sweden, a new function for coordination called Rehabilitation coordinator (RC) has been introduced within healthcare [21]. The purpose with RC, in addition to provide support to patients in their rehabilitation process [22], is to promote cooperation, streamline the sick leave and rehabilitation process, and facilitate RTW for sick-listed patients [21]. The RC’s performance has been developed over the last years, and the function is now available in primary care and to a certain extent within specialist healthcare [23]. One prioritised group for the RC is patients with mental disorders, i.e. F diagnoses, according to the International Classification of Disease (ICD) system [24].

The practice of RC’s has been developed and evaluated with positive outcomes [21, 25, 26] in the Swedish primary care. Nonetheless, to our knowledge, the effect of RC’s has not been investigated regarding reduced sick leave or RTW/entry in work or studies in patients with mental disorder within specialist psychiatric care.

This study protocol presents the design of a study aiming to evaluate a RC intervention for patients with mental disorder within the specialist psychiatry care. The objective of the study is to evaluate effects of a RC intervention on sick leave and RTW/entry in work or studies in patients with medium to severe affective and/or medium to severe anxiety disorders receiving treatment within specialist psychiatric care. The hypothesis is that the participants who receive the intervention will show a decrease in sick leave (extension and length) and to a higher degree have RTW/entry work or studies at 12-month follow-up, compared with participants receiving treatment as usual (TAU).

## Methods

### Study design and setting

The study is designed to be a randomised controlled trial (RCT) with an intervention group receiving support from a RC compared with a control group receiving TAU. The study will be conducted in specialist psychiatric care in a middle region of Sweden, between October 2018 and October 2021. The healthcare unit includes psychologists, physicians, nurses and social workers.

### Power calculation

The estimation of sample size was based on an outcome measure with two possible outcomes: proportion of patients who RTW/entry work or studies (positive outcome) and proportion of patients that has not RTW/entry work or studies (negative/unchanged outcome). The statistical programme Stata 15.0 has been used to estimate the study’s strength (statistical power). The power estimation was based on a two-sided 5% significance level. In order to detect a difference at 15% between the groups in the main outcome with around 80% power, at least 121 individuals in the intervention and the control group are needed, respectively.

### Participants and recruitments

The inclusion criteria are patients on sick leave, having moderate to severe affective and/or anxiety disorder (see Table 1 for ICD-codes), receiving treatment within a specialist psychiatric unit, aged 25–64 years, with or without employment. The exclusion criteria are multiple somatic illnesses, at high suicidal risk, fulltime sickness compensation (i.e. compensation for those between ages 30 and 64 who probably never will be able to work full time due to illness, injury or disability) [27], taking part in an ongoing vocational rehabilitation and being a patient outside of the county.

**Table 1:** Diagnostic codes in accordance to the International Classification of Disease 10-SE

Disorder	ICD code
Bipolar disorder	F31.0-F31.9
Depression	F32.1-F32.3, F33.0-F33.4, F33.9
Post-traumatic stress disorder (PTSD)	F43.1
Generalized anxiety disorder	F41.1
Neuropsychiatric disorder	F84.0, F84.1, F84.4, F84.5, F90.0

**Fig. 1:**
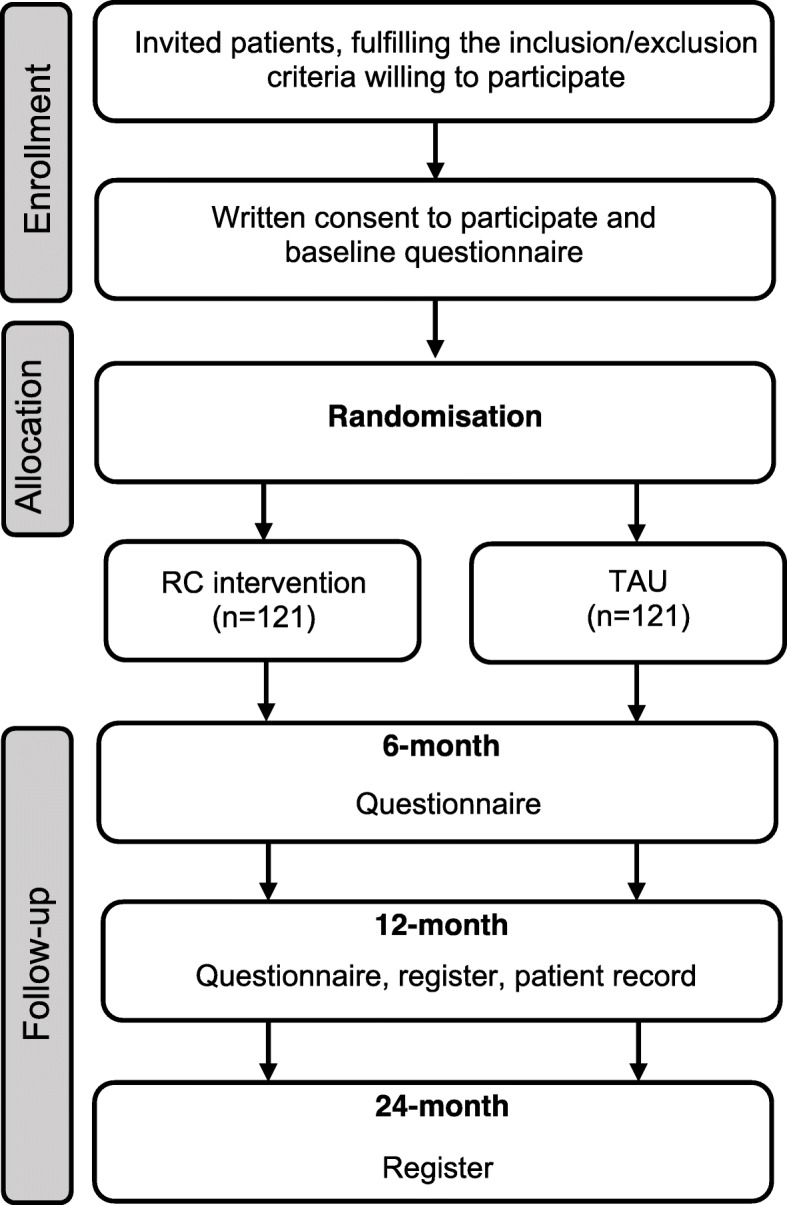
Flow chart for recruitment process and data collection

The healthcare professionals within the healthcare unit can identify prospective participants for the study. Prospective participants can also be identified by the RC through a patient register called “Rehabstöd”, which is an IT-application showing all sick listed patients in the care unit. After consulting a doctor, potential participants, fulfilling the inclusion criteria and not the exclusion criteria, will be sent an invitation letter with information about the study. After 2 weeks, a research assistant will call the potential participant and provide verbal information about the study and invite him/her to participate. If the patient is interested in participating, a consent and baseline questionnaire will be mailed to the potential participant. Those willing to participate in the study must give their written consent and answer the baseline questionnaire. Thereafter, participants will be randomised to either the intervention or control group (see Fig. 1, Flow chart for recruitment process and datacollection). Randomisation will be achieved by using a computer generated list of random numbers and will not be stratified.

### Intervention

#### Description of education and training of the RC

The RC undergoes an online course for rehabilitation coordination according to the Swedish Association of Local Authorities and Regions (SALAR). Except for an introduction about the overview of the mission and goal of the RC’s function, the course includes the following areas: *support to the patient*, *internal coordination and collaboration*, *external collaboration* and *patient cases.* Further, an online course in Insurance Medicine is obtained by the SALAR. The Insurance medicine team in the region will provide continuous competence development and process support. The RC will be given the opportunity to participate in a network together with the primary care RC’s and national network meetings for RC’s working in psychiatric care. Since RC is a new function in the specialist psychiatric care, which differs from primary care, the needs and assignments must be adapted thereto. Regular follow-up meetings will therefore take place with the process leader in the Insurance medical team to follow how the work model performs and whether something needs to be adjusted and/or clarified in the assignment.

#### Rehabilitation coordinator intervention

The RC meets the patients at the unit and assesses rehabilitation needs that are relevant for the ability to RTW/entry into work or studies, by interviewing the patient. By mapping the patient’s rehabilitation needs, a plan for the rehabilitation can be established, focusing on RTW/entry in work or studies. The RC constitutes a contact person for the participant, provides individualised support, and can initiate and coordinate contacts within- (healthcare professionals) and outside of healthcare (e.g. the SSIA, the SPES, the municipality, and the potential employer) needed for the RTW process. The RC intervention is estimated to last for approximately 1 year for patients randomised to the intervention group.

#### Strategies to improve adherence to intervention and procedures for monitoring adherence

RC’s adherence to the working method can be studied through documentation in the patient’s medical record, where the given efforts provided must be documented. The RC’s will work in accordance with the mission for RC’s described in the *Method book for coordination of sick leave and rehabilitation for healthcare* [28]. The method is organised in different levels; however, in this study, the RC will not include all these levels in the intervention. The RC’s access to the method book and contact with the process leader should be sufficient to comply with the working method.

### Control group

The control group receives TAU i.e. standard treatment at the healthcare unit and can include psychological therapy either individually or in groups, medical treatment, and psychosocial efforts. The psychosocial efforts can include support to ensure the patient’s financial security and efforts to support RTW/entry in work or studies. For description of the content in the RC intervention respectively control group, see Table 2).

**Table 2:** Description of content in the RC intervention respectively control group

RC intervention^a^	Control group
The RC - assesses the participant’s need for rehabilitation, - develops a plan for RTW/entry into work or studies together with the patient, - constitutes contact person for the participant, - provides individualised support - initiates and coordinates contacts within and outside of healthcare.	TAU can include; - psychological therapy either individually or in groups, - medical treatment - psychosocial efforts (e.g. support to ensure the patient’s financial security and efforts to support RTW/entry in work or studies)

^a^Includes TAU

### Data collection

Data will be collected from registers, medical records and questionnaires. Registry data will be collected from the SSIA’s Micro Data for the Analysis of Social Insurance Agency register (MiDAS) and the Longitudinal Integration Database for Health Insurance and Labour Market Studies (LISA) administrated by Statistics Sweden (SCB) and Easit (system for ordering information on healthcare consumption). Data regarding diagnosis (at baseline) and information about efforts/contributions and efforts by the healthcare and the RC (intervention group) will be collected through medical records, for both the intervention- and control group. Questionnaire data will be collected at baseline, 6 and 12 months. See Fig. 1 for information about the recruitment process and data collection.

### Outcome measures

The primary outcomes of the study is to evaluate effects of the RC intervention. We will analyse the difference between the intervention group and the controls in proportion of patients 1) on full- or part-time sick leave (extension) and time on sick leave (length) and 2) who RTW/entry into work or studies at 12 months.

Secondary outcomes are to perform analysis concerning workability, health outcomes and care consumption between the intervention group and the control group.

#### Registered sick leave and RTW/entry in work or studies

Primary outcomes will be measured with registry data from MiDAS and SCB. MiDAS includes information about sick leave, extension and length, and previous sick leave periods. Full- or part-time is measured as sick leave on 25/50/75% or full-time (100%) and length. LISA includes information about income (salary, remuneration via the SPES or the municipality, reimbursement/grant for studies, remuneration via the SSIA). RTW/entry into work or studies is defined as any kind of activities such as: job training, traineeships or part or full-time employment with wages or benefits, or part or full-time studies. The analyses will be performed between- and within groups 12 months after inclusion, with the possibility of a follow-up at 24 months.

### Background measure

#### Demographics

Information about educational level and country of birth and care of others (relatives or children) will be obtained through study specific questions.

#### Health related factors

Information on medical diagnosis will be collected from the patient record. General health is assessed with Self-reported health (SRH) measured with the single question ‘In general, how would you rate your health?’, answered on a five-point Likert scale from 1 = ‘Very good’ to 5 = ‘Very poor’ [29]. Health Related Quality of Life (HRQoL) is measured with the EQ-5D-3 L and includes following dimensions: mobility, personal care, anxiety/depression, everyday activities, pain/discomfort in 3 levels, where 1 = ‘No problems’, 2 = ‘Some problems’ and 3 = ‘Extreme problems’. EQ-5D-3 L also includes EQ visual analogue scale (EQ VAS), which measures self-rated health on a barometer graduated from 0 to 100 [30, 31]. The answers will be weighted and calculated to a score where 1 = full health and 0 = death. To measure pain, two questions will be used from the Örebro Musculoskeletal Pain Questionnaire [32]. The Hospital Anxiety and Depression Scale (HADS) will be used to assess anxiety and depression [33]. HADS is responded to on a four-point Likert scale from 0 to 3. The items are summed in two subscales, with scores ranging from 0 (no distress) to 21 (maximum distress). A score of 0–7 indicates a ‘non-case’, 8–10 a ‘possible case’ and 11–21 a ‘probable case’ of anxiety and depression. Psychological well-being is measured with the Questionnaire on well-being, consisting of 18 statements about how one feels feeling during the past week, answered on a five-point Likert scale from 1 = ‘Never’, 2 = ‘Rarely’, 3 = ‘Occasionally’, 4 = ‘Often and 5’ = ‘Very often’. Higher scores indicate a greater degree of perceived well-being [34]. Functioning in daily life will be assessed with the World Health Organization Disability Assessment Schedule (WHODAS) [35]. The General Self-Efficacy Scale (GSE) measures a person’s belief in their ability to handle various difficult demands in life [36]. GSE consists of 10 statements and is reported on a four-point Likert scale ranging from 1 = ‘Not at all true’ to 4 = ‘Completely true’. Consumption of care is measured through the individual question ‘Have you visited or been visited by any of the following in the last three months?’ with the following alternatives: doctor at hospital/health centre, district nurse, psychologist, social worker, physiotherapist, chiropractor/naprapath, etc., RC and been hospitalised. Answer options are: ‘No’, ‘Yes, once’, ‘Yes, several times’.

#### Social support

The following question regarding perceived emotional social support will be used: ‘Do you have anyone you can share your innermost feelings with and trust in?’ The following question will be used to assess perceived instrumental social support: ‘Can you get help from another person/other persons if you have practical problems or are ill?’ The questions were derived from Sweden’s national public health survey “Health on equal terms” [37].

#### Work related factors

Two study specific questions will be used to investigate factors related to work and employment status: ‘Did you have an employer and a job at the beginning of sick leave from which you are on sick leave?’ and ‘What is/was your main occupation or job from which you were laid off?’ The first question is answered with ‘Yes’ or ‘No’, and the second question is answered with an open answer. The single question in the Work Ability Index (WAI) [38] concerning the item “current work ability compared with the lifetime best” is used to measure self-reported work ability, firstly in relation to main occupation/job from which the person is on sick leave. The same question is used to measure work ability in relation to any kind of work. The questions are answered on a scale from 0 to 10, where 0 = ‘Completely unable to work’ and 10 = ‘Work ability as its best’. Six study specific questions will be used to investigate motivation for work. Question (1) ‘I think work will be a part of my life in the future’, (2) ‘I am motivated to start working’ and (3) ‘I am motivated to start studying’, all with answer options on a five point Likert scale from ‘Totally agree’ to ‘Totally disagree’. Question (4) ‘What chance do you think you have to be able to work in six months?’ is answered on a scale from 0 to 10, where 0 = ‘No chance’ and 10 = ‘Great chance’. Question (5) ‘Do you think that you can work in any job right now with your current health?’ and (6) ‘Do you think that you can study right now with your current health?’, both with answer options ‘Yes, absolutely’, ‘Yes, maybe’, ‘Do not know’, ‘No, hardly’ ‘No, absolutely not’. Both questions provide the opportunity to answer in which area they think they can work/study and to what extent (number of hours per week).

### Assessment questions of intervention

#### Contact with the RC

Information about the contact with the RC will be investigated in the intervention group and is measured with different study specific questions answered at different times (6 and 12 months). (1) ‘Have you participated in any activities organised by a RC in the last 6 months?’ Answer options ‘Yes’ or ‘No’. If the response is yes, then question is asked as to how many times and (2) ‘Have these meetings/activities been rewarding for you?’ answered on a scale from 0 to 10, where 0 = ‘Not at all rewarding’ and 10 = ‘Very rewarding’. Question (3) ‘Have you and your RC created a rehabilitation plan for you?’ is answered with ‘No’, ‘Yes, but it’s not done yet’ or ‘Yes, it’s done’. Question (4) ‘Has your RC been a source of support for you on the way to achieving your rehabilitation goals?’ has answer options ‘No’, ‘Yes, to some extent’ or ‘Yes, to large extent’. Question (5) ‘How has the support by the RC worked so far?’ is followed by eight claims: ‘I have been involved in my planning’, ‘I have received support in filling out forms’, ‘I have gained an understanding of my health condition’, ‘I have received the support I needed in my contact with my employer’, ‘I have received the support I needed in contact with the SPES’, ‘I have received the support I needed in contact with the SSIA’, ‘I have received the support I needed in contact with the healthcare’, ‘I have received the support I needed in contact with the municipality’. All claims will be answered with options ranging from ‘Totally agree’ to ‘Do not agree at all’ or ‘Not current/Don’t know’. (6) ‘How satisfied or dissatisfied are you with the help you received from the rehabilitation coordinator regarding: ‘Mapping of my work-/study situation’, ‘The planning of my vocational rehabilitation’, ‘Help with coordinating of my vocational rehabilitation’, ‘I have received answers to my questions’, ‘Helped me to increase my motivation to return to work/study’, ‘The interpersonal approach from the RC’, ‘I have been involved in the rehabilitation process’, ‘The RC has given me support during the sick leave process’, all answered on a five-point Likert scale ranging from ‘Very dissatisfied’ to ‘Very satisfied’.

### Data management and analysis

All participants will receive a code and data will be depersonalized. The linking code will be kept separate from the research data. All data will be storage in an electronic security-classified storage area. Measures to prevent loss of data will be taken through the use of automatic back-up routines implemented in the storage system. Access to data will be restricted to the research personnel working directly with data entry or analyses.

Intention-to-treat analyses will be performed, and if relevant, per-protocol analyses will be conducted. The main statistical analyses will be done with regression analyses, where the effect of the intervention will be measured against the respective main outcome. Differences in proportions between the intervention and control group regarding full- or part-time sick leave and RTW/entry into work or studies will be analysed with descriptive statistics and logistic regression analyses. Linear regressions will be used to analyse differences in length of time for sick leave between the groups. The regression analyses will be adjusted for potential confounders, and missing data will be handled with multiple imputations if needed. Data will be analysed using Statistical Package for the Social Sciences, SPSS, version (IBM, Corporation, Armonk, New York, USA). A significance level of *p* < 0.05 will be considered statistically significant.

## Discussion

This study aims to evaluate a RC intervention on sick leave and RTW/entry into work or studies among patients who are treated for mood and/or anxiety disorder.

A strength of this study is the randomised controlled design and the use of registry data. This study also has limitations and challenges that need to be mentioned. One limitation of the study is that participants in both the intervention and the control group cannot be blinded for the other healthcare professionals at the unit. One challenge in this study might be that involvement and support from an employer will not be possible, since approximately 50% of the patients in the healthcare unit are estimated to be unemployed and thus have no connection to a workplace. Involvement and support from the employer has shown to facilitate RTW [39], and RC efforts are usually targeted towards employed individuals on sick leave.

However, it has been found that support and collaboration (e.g. healthcare and/or a specialist) in the vocational rehabilitation are viewed as sources of support in finding an employment and also helping the individual feel encouraged in their pursuit to reach their vocational goal [40]. This applies well for the study population, especially for those without employment.

Vocational rehabilitation is carried out in cooperation with several stakeholders; therefore, this study will be dependent of welfare organisations such as the SSIA and the SPES. For those without an employer, the involvement on the SPES will be even more important. However, parallel to the study, the SPES is undergoing a major reorganisation [41], and it is unclear what impact this will have on the study’s results.

There is many negative consequences of being on long-term sick leave. In general, mental disorders generate longer periods of sick leave than somatic disorders [2]. Since mental disorders are an increasing problem, successful efforts are needed to be find to support these individuals in their sick leave and rehabilitation process. This study provides an opportunity to contribute to new knowledge in the research area. If the study has promising outcomes regarding RTW/entry into work or studies, it may be of importance for the participants in many ways (e.g. bring a sense of being active, experiencing meaningfulness [42], contribute to social relations [43] and provide economic benefits [43, 44]) and perhaps improve health and wellbeing. If so, this would be valuable for the individual and for the society, e.g. by reducing financial costs.

The results could also be relevant based on a new law in Sweden [45], promoting county councils/regions to offer coordination efforts to patients who have a special need for individual support in order to RTW/entry working life.

## Data Availability

This article describes a study protocol; thereby data sharing is not applicable as no datasets are generated or analysed.

## Abbreviations

LISA: Longitudinal integration database for health insurance and labour market studies
MiDAS: Micro data for the analysis of social insurance agency register
RC: Rehabilitation coordinator
RTW: Return to work
SALAR: Association of local authorities and regions
SCB: Statistics sweden
SPES: Swedish public employment service
SSIA: Swedish social insurance agency
